# Population Structure and Genetic Diversity of Sheep Breeds in the Kyrgyzstan

**DOI:** 10.3389/fgene.2019.01311

**Published:** 2019-12-12

**Authors:** Tatiana Deniskova, Arsen Dotsev, Eugenia Lushihina, Alexey Shakhin, Elisabeth Kunz, Ivica Medugorac, Henry Reyer, Klaus Wimmers, Negar Khayatzadeh, Johann Sölkner, Alexander Sermyagin, Asankadyr Zhunushev, Gottfried Brem, Natalia Zinovieva

**Affiliations:** ^1^L.K. Ernst Federal Science Center for Animal Husbandry, Podolsk, Russia; ^2^Institute of Biotechnology, National Academy of Science of Kyrgyz Republic, Bishkek, Kyrgyzstan; ^3^Population Genomics Group, Department of Veterinary Sciences, Ludwig Maximilians University of Munich, Munich, Germany; ^4^Institute of Genome Biology, Leibniz Institute for Farm Animal Biology, Dummerstorf, Germany; ^5^Division of Livestock Sciences, University of Natural Resources and Life Sciences Vienna, Vienna, Austria; ^6^Institute of Animal Breeding and Genetics, University of Veterinary Medicine, Vienna, Austria

**Keywords:** Kyrgyzstan, single nucleotide polymorphism, population structure, local sheep breeds, admixture, Great Silk Road

## Abstract

Sheep are a main livestock species of Kyrgyzstan, a Central Asian country with predominating mountain terrain. The current gene pool of local sheep resources has been forming under diverse climate conditions from the era of the trading caravans of the Great Silk Road, through the Soviet period of large-scale livestock improvements, which was followed by the deep crisis at the end of the 20th century, up to now. However, not much is known about the genetic background and variability of the local sheep populations. Therefore, our aims were to provide a characterization of the population structure and genetic relations within the Kyrgyz sheep breeds and to study their genetic connections with the global sheep breeds using SNP analysis. Samples of the Alai (n = 31), Gissar (n = 30), Kyrgyz coarse wool (n = 13), Aykol (n = 31), and Tien-Shan (n = 24) breeds were genotyped with the OvineSNP50 BeadChip or the Ovine Infinium HD BeadChip (Illumina Inc., USA). The measure of inbreeding based on runs of homozygosity showed a minimum value in the Aykol breed (F_ROH_ = 0.034), while the maximum was found in the Alai breed (F_ROH_ = 0.071). Short ROH segments (ROH ≤ 4 Mb) were predominant in all breeds. Long ROH segments (ROH > 16 Mb) were absent in the Gissar breed. The Gissar and Aykol breeds had the highest values of the effective population sizes estimated for five generations ago (*Ne_5_* = 660 and 563), whereas the Alai and Kyrgyz coarse wool displayed lower values (*Ne_5_* = 176 and 128, respectively). The synthetic origin of the Aykol breed was clearly evidenced by all analyses applied. Based on the network and admixture analyses of the Kyrgyz and global sheep breeds, the Tien-Shan and the Russian semi-fine wool breeds demonstrated a common ancestry that most likely is due to a contribution of the Lincoln breed. The Gissar, Aykol, and Kyrgyz coarse wool breeds showed a genetic background predominating in sheep populations from Iran and China whereas the Alai demonstrated the different ancestry type. The revealed admixture patterns probably resulted from the exchange and trade during the era of the Great Silk Road, which partly overlapped with historical and archeological findings.

## Introduction

The Kyrgyz Republic (Kyrgyzstan) is a Central Asian country comprising the Western part of the Tien-Shan and the Northern regions of the high Pamir Mountains and of the Alay Range (Raimzhanov and Lushihina, 2017). Accordingly, over 90% of the terrain is represented by mountains. Most of the valleys are situated at an altitude of 600 to 1,400 m above sea level (Schillhorn van Veen, 1995; Kadyrkulov and Kalchayev, 2000; Ibragimov and Asanaliyev, 2000). Despite the challenging landscape and shortage of arable land, Kyrgyzstan has great capacities to produce economically cheap and environmentally friendly livestock products using about 9.6 million hectares of natural mountain pastures (Lushihina, 2013).

Livestock farming (especially sheep breeding) has been the foundation supporting the livelihoods of local people from nomadic times until the present day. The contribution of the agricultural production to the economy is crucial (about 45% of gross income) (Kadyrkulov and Kalchayev, 2000). However, during the twentieth century, the sheep breeding industry underwent great changes that were caused by political and economic alterations (Kerven et al., 2011).

The traditional agricultural management and extensive mountainous land use setting in the Kyrgyzstan, which is referred to as «Central Asian mountain agro-pastoralism» (Kerven et al., 2011), was based on year-round migration cycles. The tribes practicing vertical transhumance bred local fat-rumped coarse wool sheep that were characterized by such valuable traits as high adaptation to severe mountain conditions, satisfactory meat and fat qualities, high growth energy, endurance, and resistance to various diseases (Farrington, 2005; Lushihina, 2013). Significant changes in livestock management and in the preferences of sheep breed composition took place in the Soviet period (Schillhorn van Veen, 1995; Kerven et al., 2011). Aiming to increase the wool production, large scale improvement of local sheep with highly productive breeds resulted in the creation of the Kyrgyz fine wool breed in 1956 (Ernst et al., 1994), the Tien-Shan breed with crossbred wool in 1966 (Ernst et al., 1994; Lushihina, 2013), and the Alai breed with white lustrous carpet wool in 1981 (Ernst et al., 1994; Ajibekov and Almeyev, 2008).

Furthermore, during the Perestroika and the following economic crisis, farmers intensively crossed the populations of fine wool and local coarse wool sheep with Gissar rams to obtain flocks with good meat and fat quality to overcome the food shortage in the country (Lushihina, 2013). The newly created sheep type is known as the Aykol breed (Lushihina, 2013). Over the next decades, the population number of fat-rumped coarse wool sheep including the highly productive Gissar breed (Lushihina, 2013) increased. The reasons for such a trend included the depression of the world wool market and the rising price for mutton, which is the key ration component of the Kyrgyz families (Schillhorn van Veen, 1995; Schmidt, 2001; Farrington, 2005; Kerven et al., 2011).

The sheep breed resources are among the most valuable bases of economic prosperity and food security as well as cultural heritage of the Kyrgyzstan. In this regard, an awareness of the current status of their gene pool including estimation of levels of inbreeding and an evaluation of possible admixture patterns are crucial for both the rational management of the sheep populations and the conservation of their unique traits.

The breeding of the native and locally developed livestock resources is usually based on using a restricted number of sires and mating between close relatives over generations. This process causes consecutive changes in the population means starting from an increase in inbreeding through the following reduction of genetic diversity and resulting in deterioration of fitness and survival-related traits. Furthermore, frequency of homozygous recessive defects is raised (Curik et al., 2014).

Assessment of genetic diversity based on DNA marker data is especially beneficial when pedigree records are not available or unreliable (Flury et al., 2010; Li et al., 2011; Al-Mamun et al., 2015). The development of commercial DNA arrays made multiple SNP genotyping the most useful and robust tool to infer the inbreeding level in livestock populations at genome level.

To estimate inbreeding, diverse methods are currently available. Besides well-established classical approaches such as the calculations of observed and expected heterozygosity and Wright’s F statistic indices (Fis), there are the evaluation of effective population size (Ne) based on linkage disequilibrium (LD) (Hayes et al., 2003; Corbin et al., 2010; Kijas et al., 2012) and the identification of runs of homozygosity (ROH) (Sölkner et al., 2010; Ferenčaković et al., 2011; Purfield et al., 2012). The ROH-based estimation of inbreeding is considered as most powerful approach and makes it possible to distinguish between recent and ancient inbreeding (Keller et al., 2011).

Runs of homozygosity are contiguous stretches of homozygous loci that inbred offspring inherit from both parents originated from a common ancestor (Broman and Weber, 1999; McQuillan et al, 2008; Curik et al., 2014). The length of ROH provides insight whether inbreeding was recent or ancient in a population (McQuillan et al., 2008; Curik et al., 2014), and length and distribution of ROH can be successfully used to elucidate demographic history of populations (Purfield et al., 2012; Bertolini et al., 2018).

Furthermore, it is worth mentioning that the Kyrgyzstan, which is geographically located between Kazakhstan, Uzbekistan, Tajikistan, and China, played a crucial role as a gateway of the ancient Great Silk Road (Taylor et al., 2018). In fact, the trade channels through the Kyrgyz territory, including the iconic Irkeshtam Pass, remained unaltered during the whole existence of these links between the East and the West, while the other routes changed from time to time (Williams, 2014; Abdrisaev, 2017). Along with trading of valuable goods (silk, spice, musk, tea etc.), the vast exchange of domestic animals was inevitable (Boivin, 2017; Taylor et al., 2018). To date, large amounts of SNP genotyping data have been generated from worldwide popular sheep breeds (Kijas et al., 2012; Ciani et al., 2015) and local populations including Welsh (Beynon et al., 2015), Ethiopian (Edea et al., 2017), Chinese (Zhao et al., 2017), and Russian sheep (Deniskova et al., 2018). Analysis of whole genome data on the Kyrgyz sheep breeds including available SNP profiles of the European and Asian sheep populations might be useful to establish probable admixture patterns that resulted from ancient trade interactions.

Therefore, our aims are to provide a detailed characterization of the population structure, to infer inbreeding levels and genetic relations within the Kyrgyz sheep breeds, and to study their genetic connections with the worldwide sheep breeds using the OvineSNP50K BeadChip and the Ovine Infinium HD BeadChip (Illumina Inc., USA).

## Materials and Methods

### Breeds and Samples

In total, 129 animals representing five local and locally derived breeds of the Kyrgyzstan were selected for our study including Alai (n = 32), Aykol (n = 31), Gissar (n = 30), Kyrgyz coarse wool (n = 13), and Tien-Shan (n = 24). Three of these breeds (Aykol, Gissar, and Kyrgyz coarse wool) are fat-rumped coarse wool sheep; the Alai is a fat-rumped semi-coarse wool sheep; the Tien-Shan is a long-thin-tailed sheep with crossbred semi-fine wool.

To infer the genetic relationships of the Kyrgyz sheep breeds and to study their historical admixture patterns in the context of the worldwide gene pool, we combined our data set with SNP genotypes of 61 global sheep populations, including 22 Russian, four Central European, 23 South-West European, three Asian, three Southwest Asian, and one American breeds. The sheep data made available by the International Sheep Genomics Consortium (Kijas et al., 2012) and were obtained from other publicly available sources (Ciani et al., 2015; Deniskova et al., 2018). Description of 61 sheep breeds used in the study is provided in **Supplementary Table 1**.

### DNA Extraction

DNA was extracted from ear tissue samples using Nexttec columns (Nexttec Biotechnology GmbH, Germany) according to the manufacturer’s instructions. The concentrations of the dsDNA solutions were measured with a Qubit 3.0 fluorimeter (Life Technologies, USA). The OD260/OD280 ratio of DNA solutions was determined with a NanoDrop-2000 (Thermo Fisher Scientific, Wilmington, DE, USA). Additionally, DNA quality was checked by 1% agarose gel electrophoresis.

### SNP Genotyping and Quality Control

SNP genotyping was performed using the OvineSNP50 BeadChip (Illumina, San Diego, CA, United States) or the Ovine Infinium HD BeadChip (Illumina, San Diego, CA, United States) (Kijas et al., 2014). Genotype quality control (QC) procedures were performed using PLINK v1.90 (Chang et al., 2015). To consider the accuracy and efficiency of SNP genotyping, valid genotypes for each SNP were determined by setting a cutoff of 0.5 for the GenCall (GC) and GenTrain (GT) scores (Fan et al., 2003). Samples that did not pass the quality criteria (missing genotype call rate 0.1) were excluded from the analysis.

After merging the genotypic data from the 600K and 50K arrays, a total of 42,230 autosomal SNPs that overlapped between the two arrays were left in the analysis. SNPs with a call rate below 0.90, a minor allele frequency (MAF) lower than 0.05, or that were located on sex chromosomes were discarded. A Hardy-Weinberg equilibrium test was not performed for comparisons with worldwide breeds because too many SNPs would have been excluded due to the Wahlund effect (Wahlund, 1928). After pruning 38,510 SNP remained for analysis.

To account for the effects of family structures within the subpopulations, closely related animals (relationship >0.35) were discarded based on estimation of a unified additive relationship (UAR) matrix (relationship > 0.35) according to Yang et al. (2010).

### Genetic Diversity

To estimate within-population genetic diversity, we calculated the observed heterozygosity (Ho), unbiased expected heterozygosity (H_E(u)_) (Nei, 1978), rarefied allelic richness (A_R_) and the inbreeding coefficient (*F_IS_*) based on the unbiased expected heterozygosity using the R package “diveRsity” (Keenan et al., 2013).

### Runs of Homozygosity (ROH) and Genomic Inbreeding (F_ROH_)

For estimation of runs of homozygosity (ROH), a window-free method for consecutive SNP-based detection [consecutive runs method (Marras et al., 2014)] implemented in the R package “detectRUNS” (Biscarini et al., 2018) was used. One SNP with missing genotype and up to one possible heterozygous genotype was allowed in the run. The minimum ROH length was 1000 kb.

To minimize false positive results, the minimum number of SNPs (l) was calculated as was initially proposed by Lencz et al. (2007) and followed by Purfield et al. (2012) in a study on cattle breeds

$$l=\log_{e}(\alpha /n_{s}*n_{i}))/(\log_{e}(l-het),$$

where n_s_ = the number of genotyped SNPs per individual; n_i_ = the number of genotyped individuals; α = the percentage of false positive ROH (set to 0.05 in our study); and het = the mean heterozygosity across all SNPs. Calculated l was equal to 18.

ROH were estimated for each individual and then categorized in the following ROH length classes: (1–2 Mb, 2–4 Mb, 4–8 Mb, 8–16 Mb, > 16 Mb). We computed the total number of identified ROH for each length category in each of the individuals of each breed. The mean sum of ROH was calculated by adding up the length of all ROH for each individual in the sheep populations and then the results were averaged per breed population.

The genomic inbreeding coefficient based on ROH (F_ROH_) was computed as the sum of the length of all ROH per animal as a proportion of the total autosomal SNP coverage (2.44 Gb). To measure the overall inbreeding, including recent and ancient common ancestors, we calculated the F_ROH_ based on the ROH with the minimum length of 1 Mb (F_ROH(1Mb)_). More recent inbreeding was estimated based on the ROH with minimum length of 5 Mb (F_ROH(5MB)_) as well as with minimum length of 10 Mb (F_ROH (10Mb)_).

### Effective Population Sizes

Trends of effective population size (*Ne*) were estimated from linkage disequilibrium (LD) as implemented in *SNeP* (Barbato et al., 2015). Default parameters were applied, except for the sample size correction, occurrence of mutation [α = 2.2; (Corbin et al., 2012)], and recombination rate between a pair of genetic markers according to Sved and Feldman (1973). The most recent estimate of *Ne* was taken five generations back (*Ne_5_*) to make our data comparable with previous sheep studies (Kijas et al., 2012; Beynon et al., 2015; Barbato et al., 2017). In addition, *Ne* estimates for *c* = 1 Mb (∼50 generations ago; *Ne_50_*), where *c* is the distance between the SNPs in Morgans, were calculated.

### Genetic Relationships and Population Structure

Pairwise genetic differentiation (fixation index, *F*_ST_) (Weir and Cockerham, 1984) between all pairs of sheep breeds were calculated using the R package “diversity” (Keenan et al., 2013). Using the matrix of pairwise *F*_ST_ values, Neighbor-Net graphs were constructed using SplitsTree 4.14.5 software (Huson and Bryant, 2006).

A multidimensional scaling analysis (MDS) based on pairwise identical-by-state (IBS) distances and Principal Component Analysis (PCA) were performed with PLINK v1.90 and visualized with the R package “ggplot2” (Wickham, 2009).

Genetic admixture was inferred using Admixture v1.3 software (Alexander et al., 2009) and plotted with the R package “pophelper” (Francis, 2017). Using a standard Admixture cross-validation procedure, the choice of K was based on the lowest cross-validation error compared to other K values (Alexander et al., 2009).

Inference of population splits and mixtures was performed using the TreeMix program (Pickrell and Pritchard, 2012). To set position of the root in the maximum likelihood (ML) tree produced by the TreeMix program we defined European Mouflon (n = 10) as the outgroup population. Furthermore, in alternative analyses we add one or two migration events in the ML tree to obtain the most frequently found variants with significant gene flow and ran 10 independent interactions for each event.

R version 3.3.2 was used to create the input files (R Core Team, 2018).

## Results

### General Statistics and Effective Population Sizes of the Kyrgyz Sheep Breeds

The main characteristics of the breeds are summarized in **Table 1**.

**Table 1:** Short description of the five studied sheep populations of the Kyrgyzstan.

Main characteristics	Breeds				
	Kyrgyz coarse wool	Alai	Tien-Shan	Gissar	Aykol
Period of creation	unknown	1934–1981	1950–1966	13th to 14th centuries	From 1990
	Lushikhin, 1964	Ernst et al., 1994			Lushihina, 2013
History of creation, breeds involved in the development	Indigenous	Complex crossing of local fat-rumped sheep with the Précoce and Sarajin rams	Mating local fat-rumped sheep with Précoce and then with Lincoln rams	Indigenous Central Asian sheep improved by folk selection	Crossing of local fat-rumped and local fine wool sheep with Gissar rams
	Lushikhin, 1964	Ernst et al., 1994			Lushihina, 2013
Tail type	Fat-rump	Fat-rump	Thin	Fat-rump	Fat-rump
	Ajibekov and Almeyev, 2008				
Wool type	Coarse	Semi-coarse	Semi-fine	Coarse	Coarse
	Ajibekov and Almeyev, 2008				
Main products	Meat, fat	Meat, carpet wool, skins	Meat, crossbred wool, skins	Meat, fat	Meat, fat
	Ajibekov and Almeyev, 2008				
Breeding region	Whole territory	Osh (Batken) region	Central Tien-Shan area (Naryn)	Whole territory	Issykkul region
	Lushihina, 2013	Ajibekov and Almeyev, 2008		Kerven, 2011	Farrington, 2005

The levels of genetic diversity were similar within the Kyrgyz sheep breeds, as presented in **Table 2**. The expected heterozygosity varied from 0.370 in the Alai breed to 0.392 in the Aykol breed, while rarified allelic richness ranged from 1.964 to 1.987 in the Alai and the Aykol breeds, respectively.

**Table 2:** Genetic diversity and effective population sizes within the Kyrgyz sheep breeds.

Breed	n	Ar	Ho	He(u)	Fis [CI = 95%]	*Ne_5_*	*Ne_50_*
ALAI	31	1.964	0.374	0.370	−0.011 [−0.013 to −0.009]	176	695
AYKL	32	1.987	0.393	0.392	−0.003 [−0.005 to −0.001]	563	3114
GISR	30	1.974	0.378	0.377	−0.001 [−0.003 to 0.001]	660	2945
KYCW	13	1.972	0.383	0.377	−0.014 [−0.017 to −0.011]	128	891
TNSH	24	1.982	0.395	0.391	−0.011 [−0.013 to −0.009]	259	929

Breeds: ALAI, Alai; AYKL, Aykol; GISR, Gissar; KYCW, Kyrgyz coarse wool; TNSH, Tien-Shan; n, number of individuals; Ar, rarified allelic richness; Ho, observed heterozygosity; He(u), unbiased expected heterozygosity; Fis, inbreeding coefficient based on the difference between He(u) and Ho with CI (confidence interval) = 95% (in square brackets); Ne_5_ – effective population size estimated for five generations ago, Ne_50_ – population size estimated for fifty generations ago.

The Gissar and Aykol breeds had the highest *Ne_5_* values (660 and 563) among all breeds, whereas the Alai and Kyrgyz coarse wool displayed lower values (*Ne_5_* = 176 and 128, respectively) and Tien-Shan took an intermediate position (*Ne_5_* = 259). A similar trend was recorded for the *Ne_50_* values. Thus, both the Gissar and Aykol breeds demonstrated high *Ne_50_* values around 3000, while the Alai had the minimum *Ne_50_* value (695) within the studied populations.

The *NeC* analysis identified several changes in effective population size of the studied Kyrgyz populations (**Supplementary Figures 1** and **2**). For all breeds, the peak of significantly increased *Ne* was recorded around 480 generations ago. Besides, the Gissar and Alai were characterized by several additional rising peaks around 300 and 240 generations ago.

Considering the more recent changes (up to 60 generations ago), the troughs of decline in *Ne* from 440 to 370 in the Alai and from 635 to 541 in the Tian-Shan sheep were found around 24–28 generations ago (**Supplementary Figure 1**). The changes in the historical effective population sizes of the Gissar and Aykol breeds were more obvious with alternating peaks of declines and increases. The value of effective population sizes decreased from 1020 to 436 in the Gissar and from 922 to 388 in the Aykol breeds from 10 to 3 generations ago (**Supplementary Figure 1**).

### Analysis of Runs of Homozygosity (ROH) and Genomic Inbreeding (F_ROH_)

**Figure 1:**
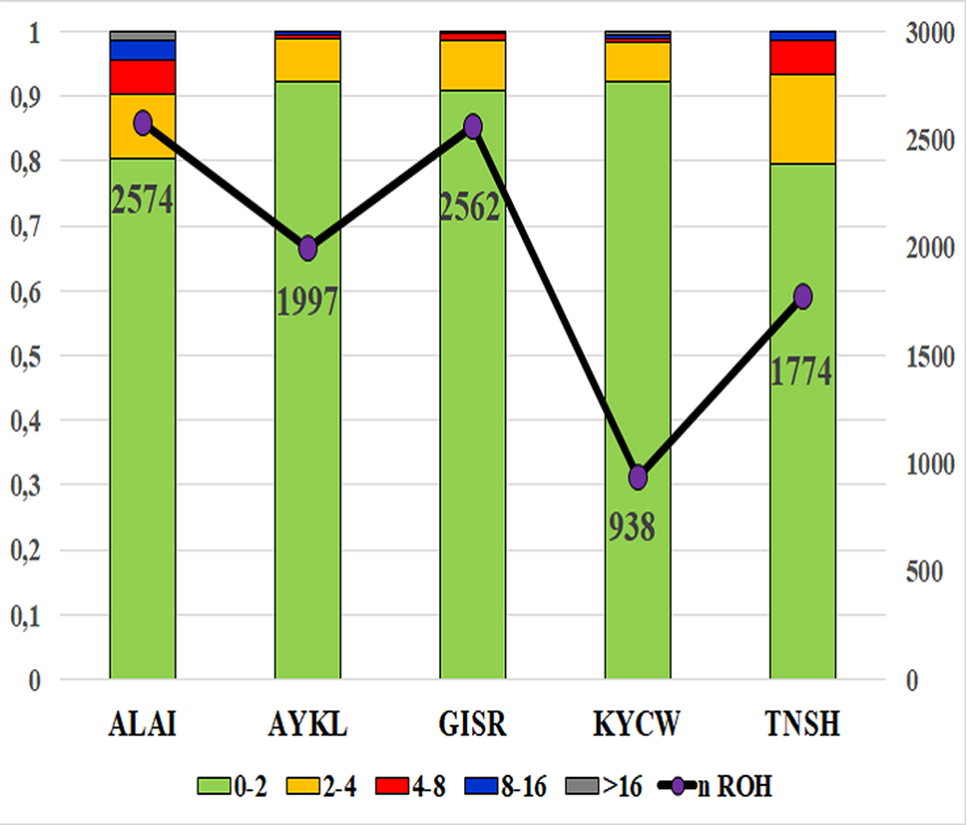
Total number of detected ROH and distribution of ROH classes among the studied Kyrgyz sheep breeds. X-axis – studied Kyrgyz sheep breeds: ALAI, Alai; AYKL, Aykol; GISR, Gissar; KYCW, Kyrgyz coarse wool; TNSH, Tien-Shan; Y-axis – distribution of ROH classes of different length; the total ROH counts in each breed are shown graphically by purple circles adjoined by digital values corresponding to the ROH numbers; the distribution ROH classes of different length is shown by a bar dendrogram: green color corresponds to ROH with a length of 0–2 Mb, orange to 2–4 Mb, red to 4–8 Mb, gray to 8–16 Mb, blue to more than 16 Mb.

ROH were found in all breeds, with short ROH segments being predominant (**Figure 1**). Thus, more than 90% of identified ROH segments in the Gissar, Aykol, and Kyrgyz coarse wool and around 80% in the Tien-Shan and Alai breeds were included in the class with the shortest length (ROH1–2Mb). The frequencies of the ROH segments of 4–8 Mb ranged from 6.18% in the Kyrgyz coarse wool breed to 13.81% in the Tien-Shan breed. The Tien-Shan and Alai breeds were characterized by a higher frequency of the ROH segments of 4–8 Mb than in the other breeds (5.24–5.40% against 0.53–0.94%). ROH segments of the long class (ROH > 16 Mb) were found in all studied breeds with exception of the Gissar breed. However, the coverage of the genome within the long ROH class (ROH > 16 Mb) was the lowest in comparison with other ROH length classes and ranged from 0.10% in the Aykol breed to 1.52% in the Alai breed (**Supplementary Tables 2**–**5**).

The Alai breed had the greatest number of ROH segments (total of 2574), while 938 ROH segments were found in the Kyrgyz coarse wool (**Figure 1**).

The lowest F_ROH_, calculated for the ROH with a minimum length of 1 Mb, was found in the Aykol breed (F_ROH (1MB)_ = 0.034), while the Alai breed had the highest F_ROH (1MB)_ with a value of 0.071 (**Table 3**). The values of F_ROH (5MB)_ ranged from 0.004 in the Gissar breed to 0.03 in the Alai breed, whereas F_ROH (10MB)_ varied from 0.005 in the Gissar breed to 0.026 in the Kyrgyz coarse wool.

**Table 3:** Summary statistics for ROH and genomic inbreeding (F_ROH_) for the studied Kyrgyz sheep breeds.

Breed	n	ROH Length			ROH Number			FROH (1MB)			FROH (5MB)			FROH (10MB)		
		Mean	Min	Max	Mean	Min	Max	Mean	Min	Max	Mean	Min	Max	Mean	Min	Max
ALAI	31	189.34±13.4	92.1	405.5	83.03±1.97	59	105	0.071	0.03	0.15	0.03	0	0.11	0.024	0	0.08
AYKL	32	89.85±2.73	63.2	154.1	62.41±1.61	41	77	0.034	0.02	0.06	0.005	0	0.02	0.009	0	0.02
GISR	30	121.83±4.13	74.1	178.1	85.4±2.36	56	114	0.046	0.03	0.07	0.004	0	0.01	0.005	0	0.01
KYCW	13	113.98±11.36	69.0	201.4	72.15±2.9	53	94	0.043	0.03	0.08	0.022	0	0.04	0.026	0.01	0.03
TNSH	24	136.73±6.89	85.0	238.5	73.92±2.38	53	99	0.052	0.03	0.09	0.01	0	0.03	0.008	0	0.02

Breeds: ALAI, Alai; AYKL, Aykol; GISR, Gissar; KYCW, Kyrgyz coarse wool; TNSH, Tien-Shan; n, number of individuals; F_ROH (1MB)_ = inbreeding coefficient calculated based on ROH with a minimum length of 1 Mb; F_ROH (5MB)_ = inbreeding coefficient calculated based on ROH with a minimum length of 5 Mb; F_ROH (10MB)_ = inbreeding coefficient calculated based on ROH with a minimum length of 10 Mb; Min – minimum values of estimations observed in individual animals within breeds; Max – maximum values of estimations observed in individual animals within breeds.

### Population Structure and Genetic Relations of the Sheep Breeds of the Kyrgyzstan

**Figure 2:**
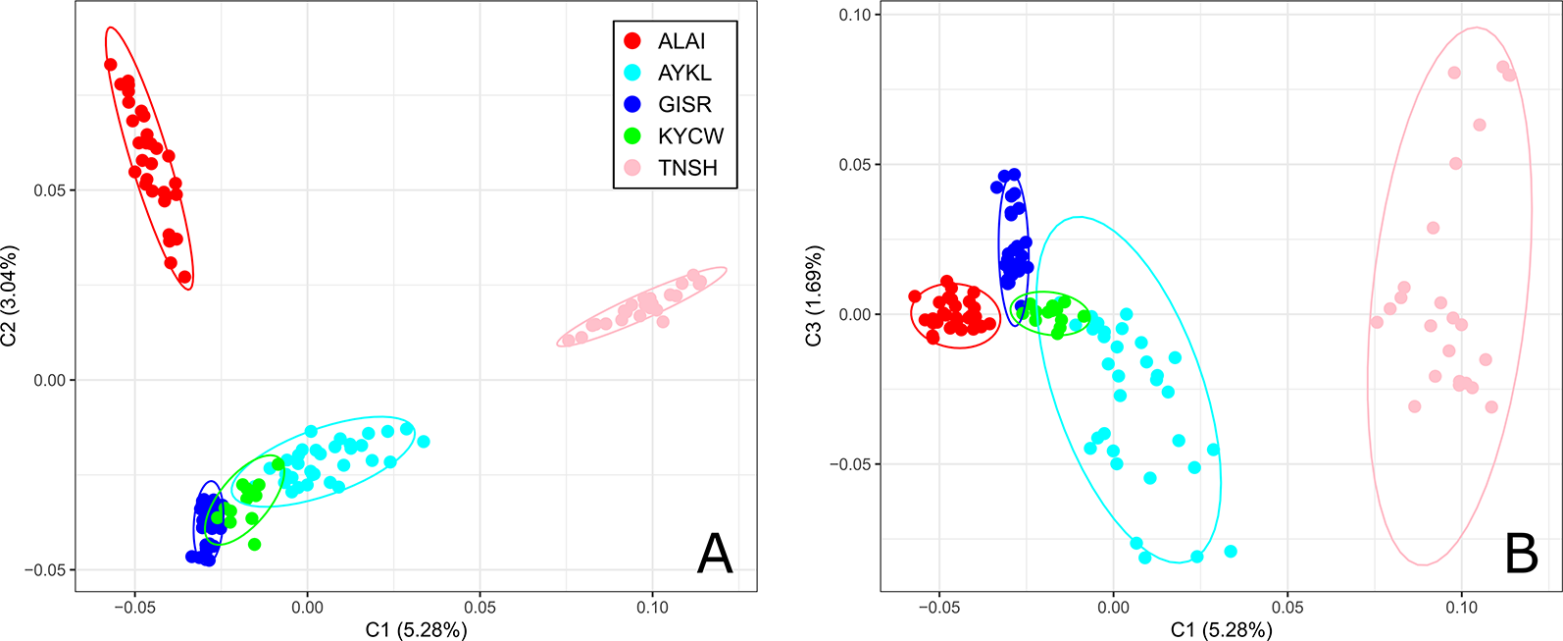
Multi-dimensional scaling analysis of the Kyrgyz sheep breeds. The analysis was performed for the first two components (C1 and C2) **(A)** and for the first and third component (C1 and C3) **(B)**. The color indications for the breeds are as follows: Alai = red, Aykol = turquoise, Gissar = blue, Kyrgyz coarse wool = green, Tien-Shan = pink. Breeds: ALAI, Alai; AYKL, Aykol; GISR, Gissar; KYCW, Kyrgyz coarse wool; TNSH, Tien-Shan.

The first component of the MDS analysis (**Figure 2**) accounted for 5.28% of the genetic variability and separated the Tien-Shan breed from the Alai, Kyrgyz coarse wool, and Gissar breeds. The Aykol breed was found at an intermediate position between the distant Tien-Shan and the fat-rumped breeds. A better understanding of such a spatial distribution of the Aykol breed was provided by the third component. The second component (with 3.04% of the genetic diversity) discriminated the Tien-Shan and Alai breeds from the remaining sheep groups. At the same time, the third component demonstrated the closeness of the Alai breed to the Kyrgyz coarse wool, Gissar, and Aykol breeds.

**Figure 3:**
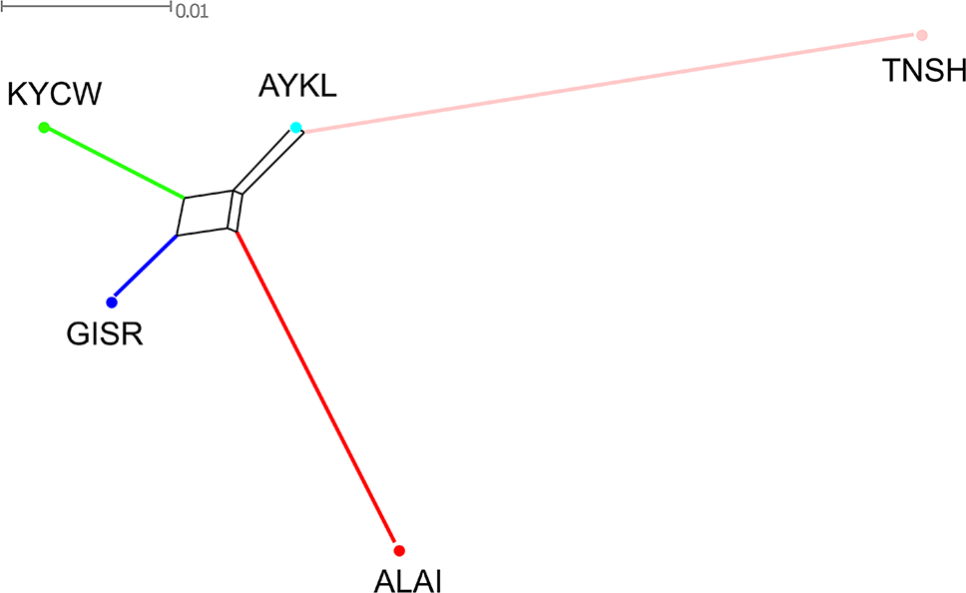
Neighbor network constructed from pairwise *F*_ST_ values for five Kyrgyz sheep breeds. The color indications for the breeds are as follows: Alai = red, Aykol = turquoise, Gissar = blue, Kyrgyz coarse wool = green, Tien-Shan = pink. Breeds: ALAI, Alai; AYKL, Aykol; GISR, Gissar; KYCW, Kyrgyz coarse wool; TNSH, Tien-Shan.

The Neighbor-Net graph (**Figure 3**) was in accordance with the MDS findings. The Alai, Kyrgyz coarse wool, and Gissar breeds formed a cluster, while the Tien-Shan was represented by a long branch on the opposite side of the net. The Aykol breed formed an extremely short branch and was positioned between the Tien-Shan breed and the cluster of the remaining breeds.

The relationships between the studied sheep breeds were assessed by calculating a pairwise *F*_ST_ matrix (**Supplementary Table 6**). The largest computed *F*_ST_ values were found between the Tien-Shan and Alai, Tien-Shan and Gissar, Tien-Shan and Kyrgyz coarse wool breeds and were 0.066, 0.057, and 0.055, respectively. The minimum *F*_ST_ value was found between the Aykol and Gissar breeds (Fst = 0.013). The pairwise *F*_ST_ values between the Aykol breed and the other breeds varied from 0.017 with the Kyrgyz coarse wool breed to 0.029 with the Alai and 0.035 with the Tien-Shan breed. The *F*_ST_ values between the Alai and Gissar breeds and the Alai and Kyrgyz coarse wool breeds were similar (*F*_ST_ = 0.030 and 0.036, respectively).

**Figure 4:**
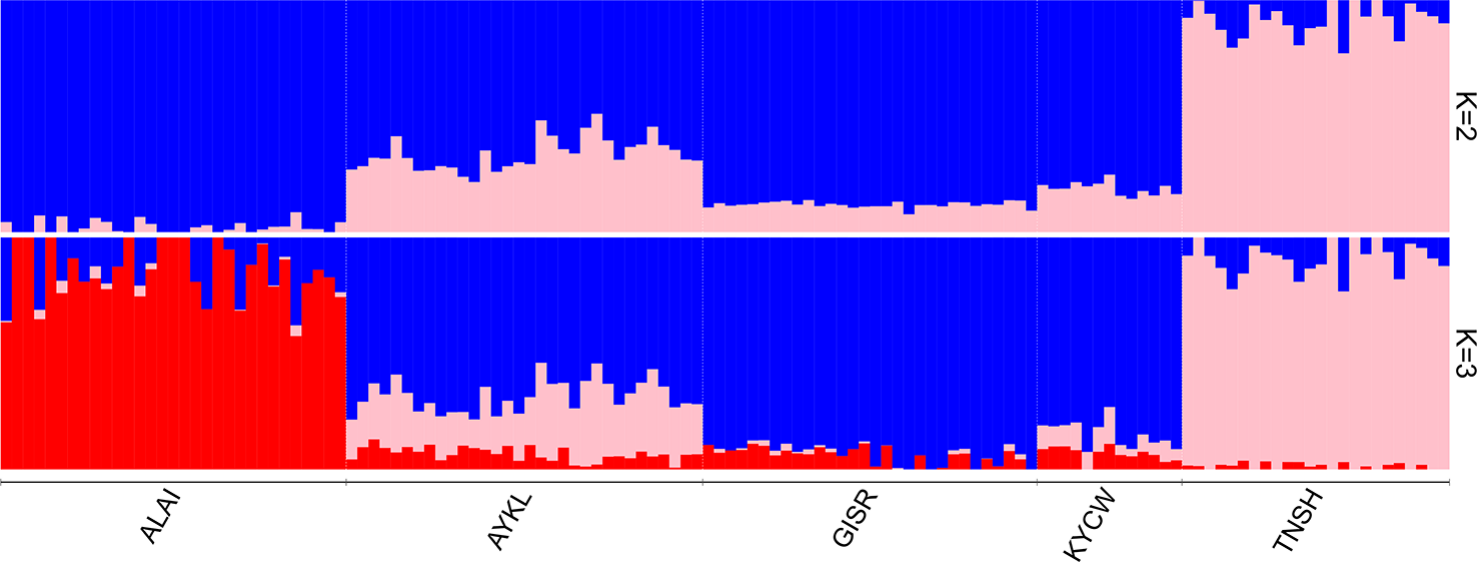
Clustering of individual animals for the Kyrgyz dataset using genome-wide SNP data. Results are shown for modelled ancestral populations K = 2–3. K = 3 was suggested as the most likely number of clusters. Breeds: ALAI, Alai; AYKL, Aykol; GISR, Gissar; KYCW, Kyrgyz coarse wool; TNSH, Tien-Shan.

The ADMIXTURE analysis (**Figure 4**) provided additional evidence of the distinctness of the Tien-Shan breed (pink, **Figure 4**) from the remaining ones at a K = 2. At K = 3, which was suggested as the most likely number of clusters (**Supplementary Figure 3**), the Alai (red) and Tien-Shan (pink) had their own clusters. The Gissar and Kyrgyz coarse wool breeds shared a common pattern of genetic ancestry (blue, **Figure 4**). The Aykol breed demonstrated an admixed origin.

### Sheep Breeds of the Kyrgyzstan in the Global Context

**Figure 5:**
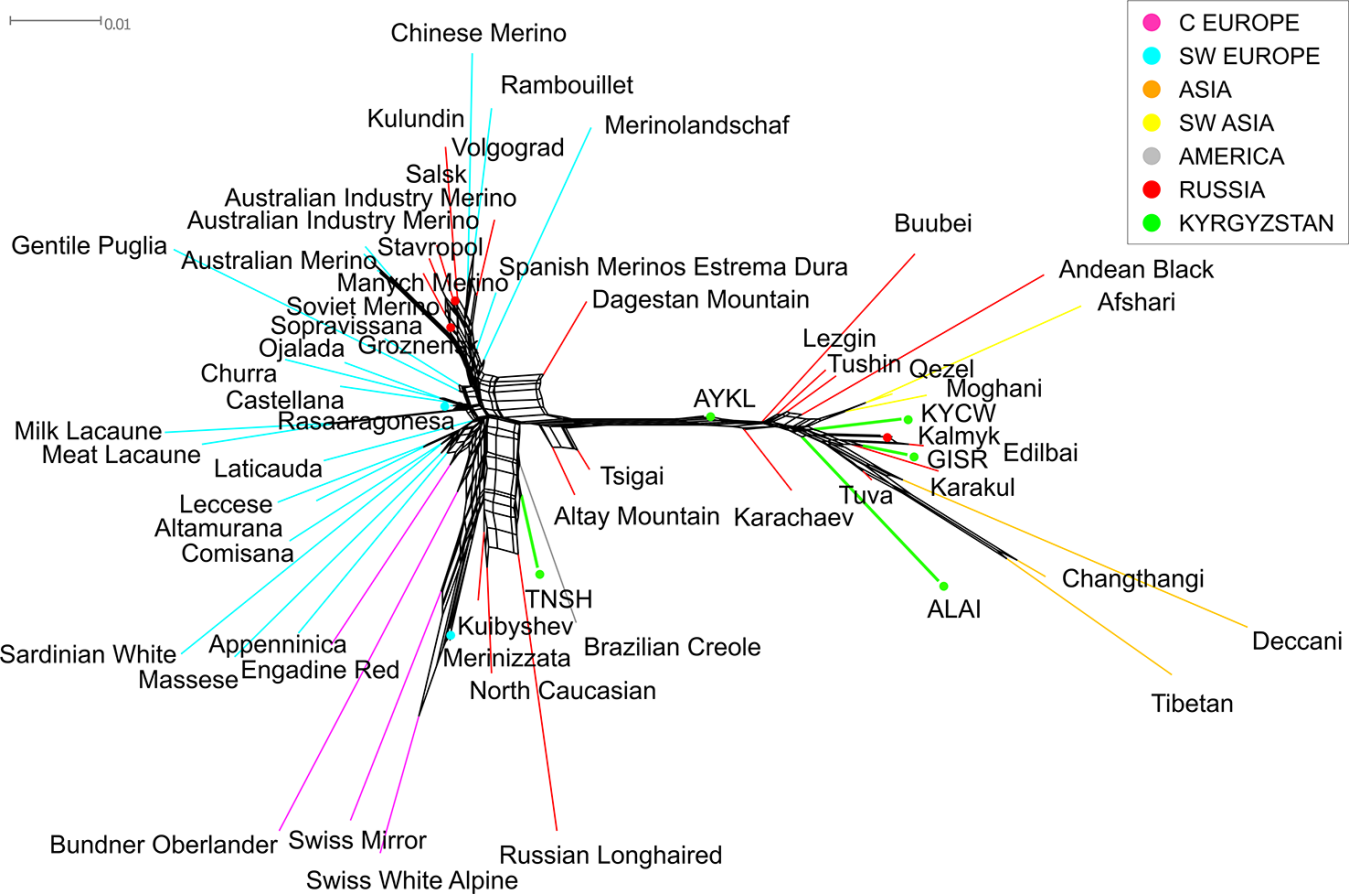
Neighbor network based on the matrix of pairwise *F*_ST_ values constructed for the combined dataset of SNP genotypes of the Kyrgyz, Russian and worldwide sheep breeds. The branches corresponding to the Kyrgyz breeds are indicated in green color. The colors of the branches of the worldwide breeds correspond to their ancestral geographic origin and are identical to the colors in **Supplementary Table 1**: pale pink for Central Europe, cyan for Southwestern Europe, orange for Asia, yellow for Southwestern Asia, gray for the Americas and red for Russia. For a description of the sheep breeds, [see **Table 2** and **Supplementary Table 1**].

The Neighbor-Net analysis including many breeds world-wide (**Figure 5**) confirmed the existence of two different ancestry patterns in the sheep breeds of Kyrgyzstan. Primarily, all breeds from the global dataset divided into two big groups corresponding to their origin (predominantly European or Asian). Accordingly, the Tien-Shan breed joined the cluster formed by several Russian (Russian Longhaired, Kuibyshev, and North Caucasian) and Central European breeds (Swiss White Alpine Sheep, Swiss Mirror Sheep).

The Alai, Kyrgyz coarse wool, and Gissar breeds were incorporated in the Asian cluster that comprised Chinese, Indian, Iranian, and Russian coarse wool breeds. The Aykol breed was located almost on the edge of the net and took an intermediate position between the two large clusters.

The Principal Component Analysis of the Kyrgyz sheep breeds in the global context (**Supplementary Figure 4**) supported the findings based on the results of the network analysis (**Figure 5**). The sheep breeds of European origin were clearly differentiated from the breeds of Asian and African origin by the first principal component accounted for 40.8% of the total variation. The first of the listed above cluster comprised the Tien-Shan breed as the second one included the other Kyrgyz sheep breeds. The intermediate position of the Aykol breed provided by the network analysis was evidently demonstrated by the PCA as well.

**Figure 6:**
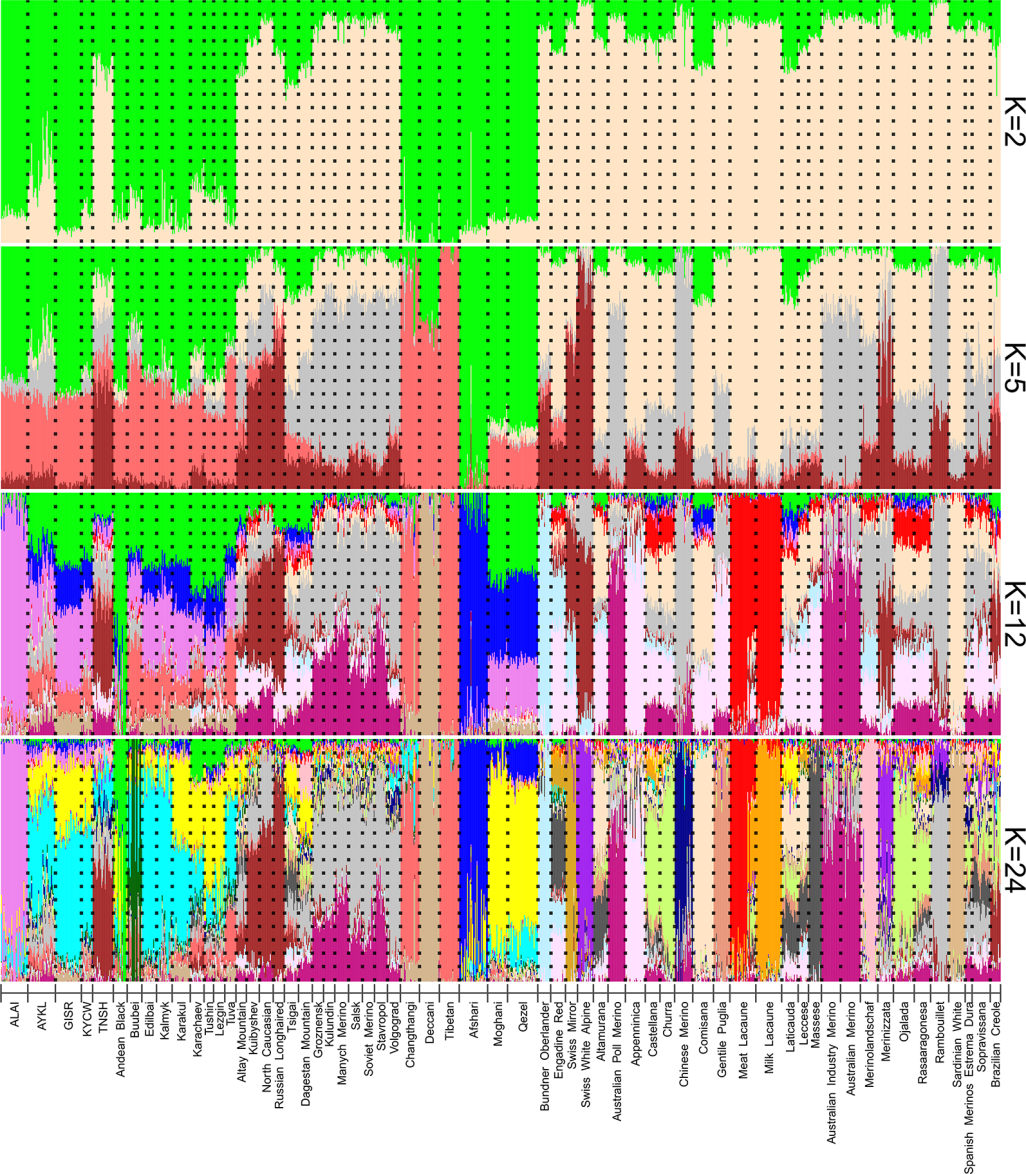
Clustering of the Kyrgyz sheep breeds in the context of the dataset of worldwide and Russian sheep breeds using genome-wide SNP data. Results are shown for modelled ancestral populations K = 2, 5, 12, and 24. K = 24 was suggested as the most likely number of clusters. For a description of the sheep breeds, [see **Table 2** and **Supplementary Table 1**].

The ADMIXTURE analysis revealed a complex genetic background of the Kyrgyz sheep breeds (**Figure 6**). According to the cross-validation error (**Supplementary Figure 5**), the most probable number of ancestral populations was 24 in the studied dataset. However, we additionally analyzed admixture patterns obtained at K = 2, 5, and 12 and contributed to a better understanding of the ancestry of the Kyrgyz breeds. The genetic pattern at K = 2 was highly consistent with the Neighbor-Net graph. The Alai, Gissar, Kyrgyz coarse wool, and Aykol breeds shared a common pattern of Asian genetic ancestry with both global and Russian coarse wool breeds (green color). The contribution from sheep populations of European origin predominated in the Tien-Shan breed at this K-value (antique white color). At K = 5, the Kyrgyz sheep populations (with exception of the Tien-Shan) demonstrated obvious shared ancestry with breeds from China (Tibetan, Changthangi) and India (Decani) (followed by prevalence of salmon color) as well as with Iranian breeds (Afshari, Qezel, Moghani) (followed by prevalence of green color). The same pattern was also obtained for the Russian fat-tailed and fat-rumped breeds with coarse wool (the columns from Buubei to Tuva). At K = 12, the Alai breed showed a different genetic background (violet color) that was found in smaller proportions in the Aykol, Gissar, Kyrgyz coarse wool as well as in the Qezel and Moghani breeds of Iran, and in the Russian coarse wool breeds (the row from Buubei to Tuva). Finally, at K = 24, the Alai contribution (violet color) was identified in the Aykol, Gissar, and Kyrgyz coarse wool breeds, which showed the obvious closeness with Iranian sheep and comprised the small shared ancestral components with Chinese and Indian breeds. The Tien-Shan breed demonstrated shared ancestry with the Russian semi-fine wool breeds including the Kuibyshev, North Caucasian and Russian Longhaired breeds (brown color). In addition, the ancestral Merino component accounted for by Rambouillet (gray color) was present in the Aykol and Tien-Shan breeds.

**Figure 7:**
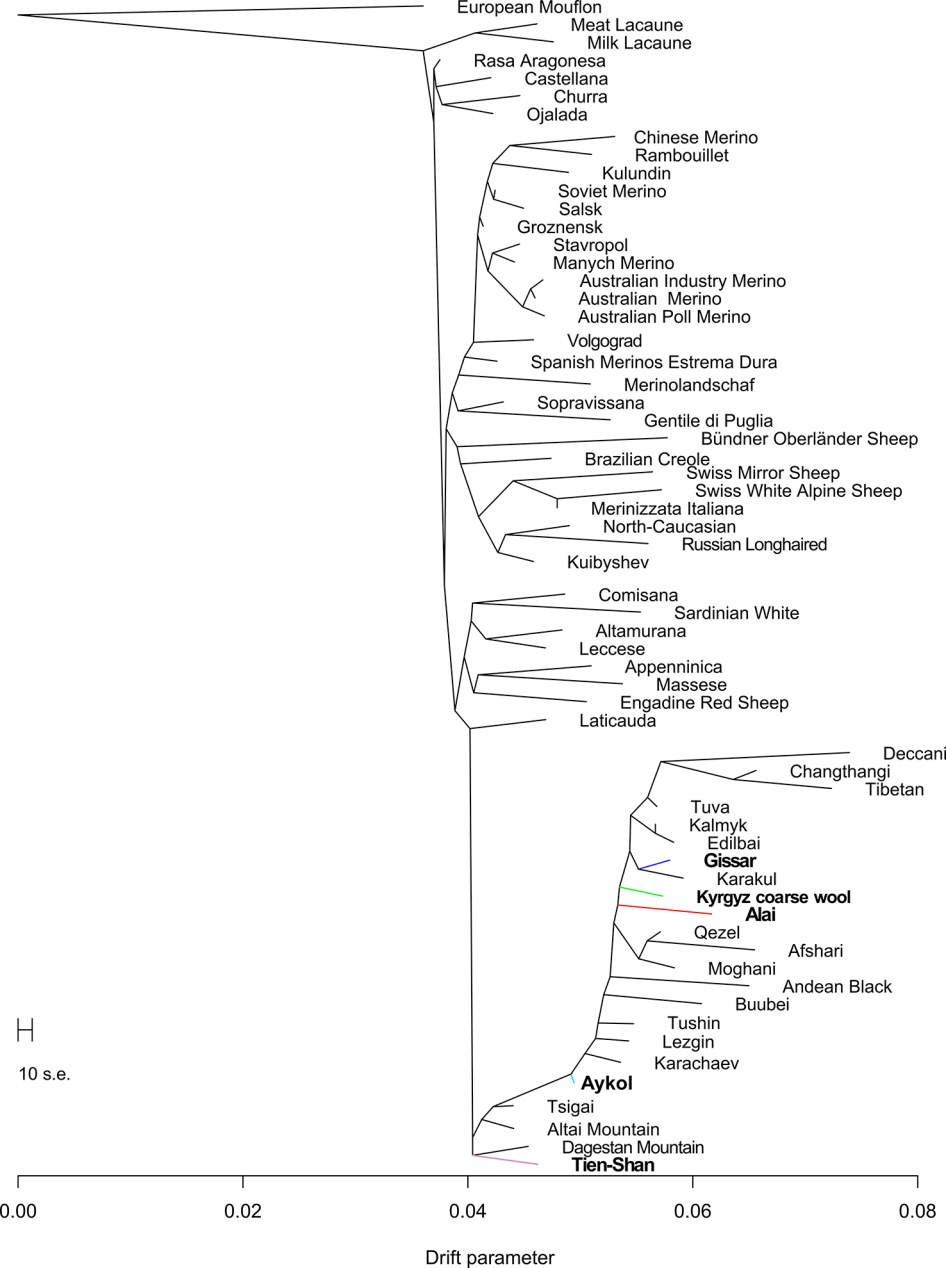
Phylogenetic network inferred by Treemix plot of the relationships between the Kyrgyz breeds and worldwide sheep populations with no migration events. The names of the Kyrgyz breeds are shown in bold. The color indications for tree branches of the Kyrgyz sheep breeds are as follows: Alai = red, Aykol = turquoise, Gissar = blue, Kyrgyz coarse wool = green, Tien-Shan = pink.

Maximum likelihood assessment of population history based on Treemix analysis with no migration events (**Figure 7**) generally confirmed aspects that have been already detected by Neighbor-Net analysis (**Figure 5**). Particularly, the Kyrgyz sheep populations with fat rumps including the Alai, Kyrgyz coarse wool, and Gissar breeds comprised the relevant cluster of coarse wool fat-tailed (or fat-rumped) sheep of Chinese, Indian, Iranian, and Russian origin. The position of the Aykol breed was transitional between the fat-tailed cluster described above and the group of thin-tailed sheep with fine and semi-fine wool. The Tien-Shan breed clustered with Russian breeds with semi-fine wool (Tsigai and Altai Mountain) and fine wool (Dagestan Mountain).

**Figure 8:**
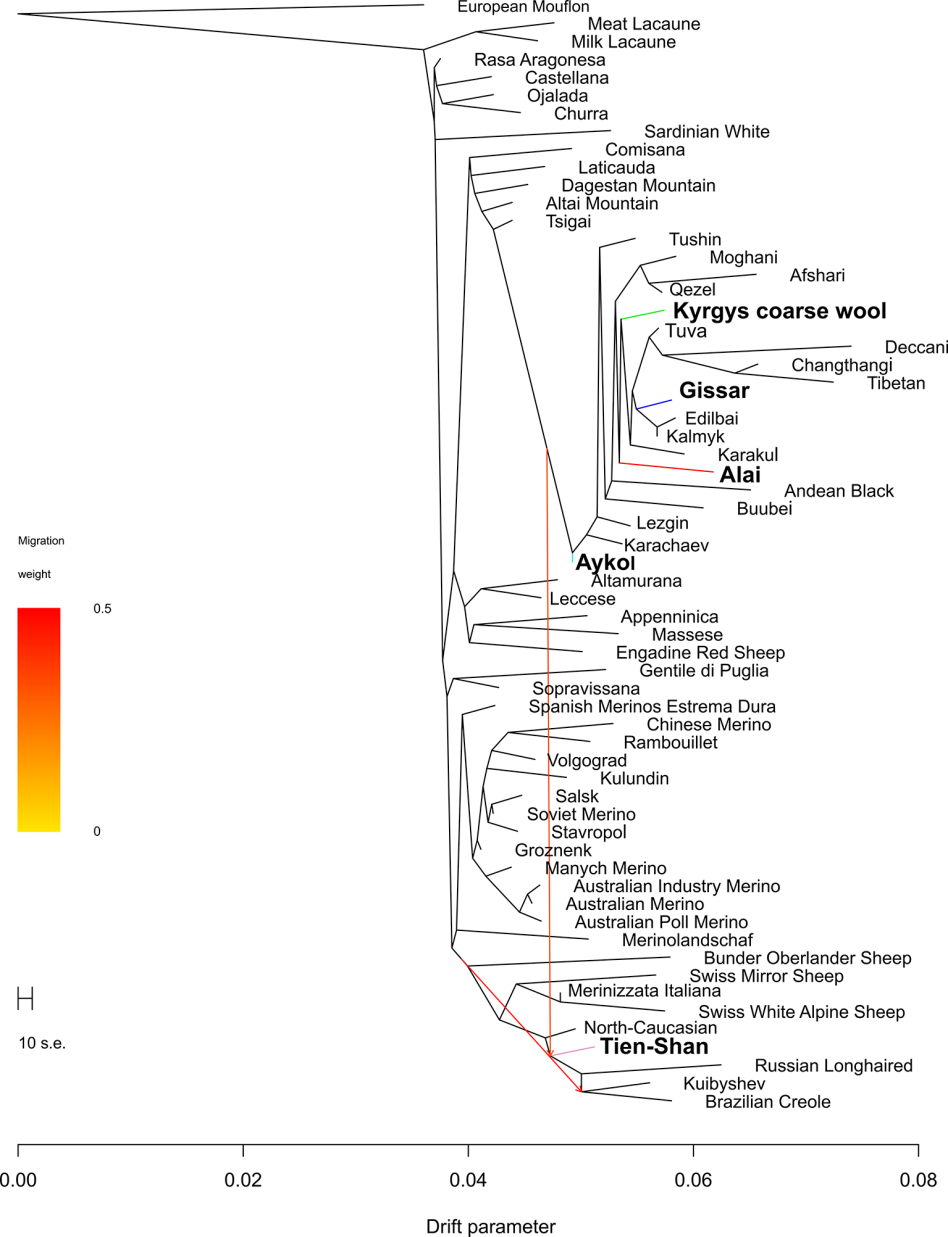
Phylogenetic network inferred by Treemix plot of the relationships between the Kyrgyz breeds and worldwide sheep populations with two migration events. The names of the Kyrgyz breeds are shown in bold. The color indications for tree branches of the Kyrgyz sheep breeds are as follows: Alai = red, Aykol = turquoise, Gissar = blue, Kyrgyz coarse wool = green, Tien-Shan = pink.

With addition of migrations events, significant replicable effects were revealed when two migration events were allowed (p < 0.05) (**Figure 8**). The first mixture event was revealed from the cluster of coarse wool fat-tailed (or fat-rumped) sheep populations to the Tien-Shan breed. The second mixture event was found from the branches of the Merinolandschaf and the crossbred breeds to Brazilian Creole breed through the Tien-Shan breed.

## Discussion

Sheep is a main livestock species of Kyrgyzstan, a Central Asian country composed by mountain regions. During its long history, the gene pool of Kyrgyz sheep breeds has undergone great changes influenced by a complex of historical and natural factors. Until now, the genetic variation of sheep resources of Kyrgyzstan has only been assessed using microsatellites (Deniskova et al., 2017; Selionova et al., 2018). In this study, we aimed to address the genetic variability and population structure within five sheep breeds in the Kyrgyzstan on a genome-wide level as well as to infer the phylogeny and to detect expected admixture patterns of these breeds in a worldwide context.

### Genetic Diversity, Effective Population Sizes and Genome Inbreeding Based on Runs of Homozygosity (ROH)

One of the points of this study was to evaluate the genetic diversity of the popular sheep breeds of the Kyrgyzstan. Our results showed that the level of genetic variability in the Kyrgyz breeds was comparable to that demonstrated in other worldwide populations (Kijas et al., 2012; Beynon et al., 2015; Ciani et al., 2015; Deniskova et al., 2018).

In general, the studied populations of the Kyrgyz breeds were characterized by high historical effective population sizes (**Table 1**, **Supplementary Figures 1** and **2**). Even the smallest values of the effective population sizes estimated for five generations ago for the Kyrgyz coarse wool and Alai breeds, for example, were higher than those reported by Purfield et al. (2017) for the Beltex breed (*Ne_5_* = 115) and by Deniskova et al. (2018) for the Kuchugur breed (*Ne_5_* = 65). Effective population sizes estimated for 50 generations ago for the studied populations were higher (for example, *Ne_50_* = 467 in the Spanish Churra breed (García-Gámez et al., 2012) or *Ne_50_* = 2171 in the Russian Karakul breed (Deniskova et al., 2018)) or in agreement with the observations reported by the SheepHapMap project (Kijas et al, 2012).

The overall significant larger effective population sizes (**Table 1**) and the several peaks of increase of the *Ne* values (**Supplementary Figures 1** and **2**) as well as low F_ROH_ values (4.6% and 3.4%, respectively) in the Gissar and Aykol breeds probably corresponded to the wide spreading and high popularity of this type of the fat-rumped breed in Central Asian countries (Karakulov, 2008; Kerven et al, 2011; Lushihina, 2013).

The low values of effective population size in the Alai as well as the moderately small values in the Tien-Shan breed may reflect the small founding populations. Both breeds were created and reared within the scientifically designed breeding programs in the Soviet period (Ernst et al., 1994; Ajibekov and Almeyev, 2008; Lushihina, 2013). There is very contradicting data on the current state of the gene pool of these breeds. Thus, according to Ajibekov and Almeyev (2008), the Tien-Shan breed includes four bloodlines derived from specific rams. In the early 2000s, around 1500 animals were subject to genealogical control (Ajibekov and Almeyev, 2008). Lushihina (2013) indicated a significant decline in population size of the purebred Tien-Shan breed and on the possible crossing with other breeds. Considering a moderate value of F_ROH_ (5.2%) of the Tien-Shan breed, we could assume that some rotation of rams takes place between the bloodlines within the breed to prevent inbreeding depression.

The current situation with the Alai breed is even more ambiguous. In the 1980s, there were five different intra-breed bloodlines developed through selection and moderate inbreeding (Ajibekov and Almeyev, 2008). Currently, breeding work with the Alai breed is conducted on private farms (Ajibekov and Almeyev, 2008) and, according to Lushihina (2013), the gene pool of the purebred Alai sheep is almost lost. These observations agreed with the low values of effective population size calculated in our study. For the present study, the most homogenous individuals of the Alai sheep in their appearance and performance were collected. Thus, the highest estimated value of F_ROH_ (7.1%) and the longest mean ROH segments (2.28 Mb) in the Alai breed seemed to be plausible. Summing up, the Alai breed is probably in critical state.

Based on the estimated low effective population size, the Kyrgyz coarse wool sheep is another valuable breed that could be in danger of extinction and is required to be included in special conservation programs (Lushihina, 2013). Nevertheless, the Kyrgyz coarse wool sheep was characterized by a low F_ROH_ value (4.3%). This pattern is in accordance with the fact that crossbreeds of the Kyrgyz coarse wool sheep and various breeds frequently form shared herds with purebred animals (Lushihina, 2013).

In general, ROH length distribution, e.g. the predominance of short ROH segments (**Figure 1**), was in agreement with the results previously obtained in different sheep populations (Al-Mamun et al., 2015; Mastrangelo et al., 2017; Purfield et al., 2017).

Considering the different methods of estimating genetic diversity (F_ROH_, Fis, *Ne*), we found no strong traces of recent inbreeding in the studied populations of the Kyrgyz sheep. We have a few assumptions on why this pattern arose. According to Kerven et al. (2011), after the split of the USSR, the management system of sheep breeding in the Kyrgyzstan has returned to traditional livestock practices. Natural mating system prevails over artificial insemination. In addition, sheep herds that belonged to different farmers possibly intermixed and intercrossed.

### History of Creation of the Kyrgyz Sheep Populations

Considering the results of the MDS (**Figure 2**), PCA (**Supplementary Figure 4**), network (**Figure 3**), and cluster (**Figure 4**) analyses, we observed a consistent separation of the Tien-Shan breed from the other Kyrgyz sheep. Maximum likelihood assessment based on TreeMix analysis with two migration events provided the detailed insight into population history of the Tien-Shan sheep (**Figure 8**) and corresponded to the origin of the breed. The Tien-Shan breed was created by crossing fat-rumped ewes with semi-coarse and semi-fine wool with the Précoce breed and with a subsequent improvement by Lincoln rams (the first mixture event) (Ernst et al., 1994; Ajibekov and Almeyev, 2008; Lushihina, 2013). The breed was developed to produce both meat and wool under the severe continental climate and high humidity of the Central Tien-Shan Mountains (Lushihina, 2013). The Neighbor-Net (**Figure 5**), admixture graphs (**Figure 6**) as well as the relevant pairwise *F*_ST_ values (**Supplementary Table 6**) demonstrated a common ancestry between the Tien-Shan and Russian semi-fine wool breeds, especially the Russian Longhaired. Most likely, this shared pattern of genetic background was accounted for by the Lincoln contribution to both breeds (Sel’kin and Sokolov, 2002). The moderate proportions of the Württemberger (Merinolandschaf) (pale pink, **Figure 6**) and Rambouillet genetic patterns (pale gray, **Figure 6**) were most likely accounted for by the Précoce origin and indirectly confirmed by the second mixture event (**Figure 8**). In addition, Nikolaev (1964) suggested that the Württemberger (Merinolandschaf) rams was used to improve the fine-wool and semi-fine wool sheep breeds inhabiting mountains regions of Russia and Kyrgyzstan.

The synthetic origin of the Aykol (Aikolskaya) breed was clearly evidenced by all analyses. The Aykol’s position in the global network (**Figure 5**) and on the PCA plot (**Supplementary Figure 4**) most likely corresponded to a lack of unique gene pool. According to Lushihina (Lushihina, 2013), the Aykol breed was developed by crossing of Kyrgyz fine-wool and local coarse-wool ewes with Gissar rams in the last decades of the 20th century. However, the observed admixture pattern (**Figure 6**) as well as specific position at the maximum likelihood trees (**Figures 7** and **8**) provided evidence that the fine wool genetic background was still present in the Aykol sheep. That most likely corresponded to the ancestry of Précoce (Ernst et al., 1994; Lushihina, 2013) and Rambouillet (Nikolaev, 1964), which had been involved in the creation of the Kyrgyz fine wool breed.

Definite genetic closeness of the Kyrgyz coarse wool and Gissar breeds was revealed by a low *F*_ST_ value (*F*_ST_ = 0.017) (**Supplementary Table 6**), joint clustering in the Neighbor-Net graph (**Figure 3**) and similar admixture patterns (**Figures 4** and **6**). At the beginning of the 20th century, the fat-rumped Kyrgyz coarse wool breed was the only known indigenous breed reared on the territory of Kyrgyzstan (FAO, 2004; Ajibekov and Almeyev, 2008; Lushihina, 2013). Being the most valuable fat-rumped breed in the Central Asian countries (Karakulov, 2008; Lushihina, 2013), the Gissar (Hissar, Gissarskaya) sheep is not native to the Kyrgyzstan. Most likely, the breed was created by long selection by Uzbek nomads, which had moved to Tajikistan with their sheep flocks in the 13^th^ to 14^th^ centuries (Ernst et al., 1994). As Ivanov and then Azarov pointed out, the Gissar breed represented «an isolated race of fat-rumped sheep» (Ivanov, 1964; Ernst et al., 1994). The Gissar breed is characterized by the outstanding adaptation to various ecological and geographical conditions including both high and low altitudes (Karakulov, 2008). The Gissar sheep was used as improving breed to increase live weight and sizes of the fat rump in the Kyrgyz coarse wool breed (FAO, 2004), which is in agreement with our findings.

The creation of the Alai (Alaiskaya) breed was a multistep process and lasted from 1934 to 1981 (Ernst et al., 1994). The ewes of the local fat-rumped sheep traditionally raised in the Alai Valley were crossed with the Précoce breed. In 1962, Sarajin rams from Turkmenistan were mated with the obtained crossbreds (Ernst et al., 1994; Ajibekov and Almeyev, 2008; Lushihina, 2013). Further selection and rigid culling of the created animals resulted in the modern Alai breed that meets high levels of productivity (meat, fat, and white carpet wool) and good adaptations to the extreme altitudes and poor fodder conditions of the Alai Valley (Ernst et al., 1994). The revealed position of the Alai breed within the other sheep groups in the Kyrgyzstan was peculiar. Evidence of the common ancestry of the Alai, Gissar, Aykol, and Kyrgyz coarse wool was provided by the admixture analysis (K = 2, **Figure 4**; K = 5, 12, and 24, **Figure 6**; **Figure 7**). However, with increasing K-values, the Alai breed demonstrated a different pattern of admixture (K = 3, **Figure 4**; K = 12, 24, **Figure 6**). In addition, the presence of the Précoce background was undetectable in the Alai group in comparison with the Tien-Shan and Aykol breeds (**Figure 6**). Furthermore, it is worth mentioning that the separation of the Alai breed occurred later than the Tien-Shan (**Figure 2**).

### The Relationships of the Kyrgyz Sheep Populations to Global Sheep Breeds

Analyzing the combined dataset of the SNP genotypes of the Kyrgyz, Russian, and worldwide breeds, the genetic background present in sheep from Iran (Moghani, Qezel, Afshari) and China (Changthangi, Tibetan) as well as in Indian Deccani was found in the Alai, Gissar, Aykol, and Kyrgyz coarse wool breeds (**Figures 5**–**8**). In our previous study, we detected that Russian local coarse wool breeds were characterized by a similar genetic pattern of admixture with the same listed breeds of Asian and Southwestern Asian origin (Deniskova et al., 2018). We assumed that this probably resulted from the nomad expansions (Yunusbayev et al., 2015), Mongol invasions (Zhao et al., 2017) as well as the trade routes defined as the Great Silk Road.

In the current study, the assumption of an influence of the Great Silk Road on the gene pool of local sheep seems to be plausible. Due to a specific geographical location and mountain landscape, the territory of modern Kyrgyzstan was the crossroad of the ancient civilizations and great empires involved in the world’s famous exchange networks connecting China and South Asia with Central Asia and Europe (Williams, 2014; Spengler, 2015; Stevens et al., 2016; Taylor et al., 2018). Based on historical records, it is generally considered that the transcontinental trade channels had formed in the first millennium BCE (Christian, 2000). However, several archaeological investigations provided evidence of possible earlier connections between the relevant countries during the Bronze and Early Iron Ages (Frachetti et al., 2017; Spengler, 2015; Boivin, 2017). In any case, the significant role of the Kyrgyz Republic as a mountain corridor for the caravans has been investigated in detail and described in different aspects (Frachetti et al., 2017; Spengler, 2015; Stevens et al., 2016). The most significant passes were the iconic Irkeshtam Pass, which was the shortest route between the Ferghana valley and Kashgar through the Alai Valley; the pass connecting Aksu and Taraz through the Lake Issyk Kul basin and the Chu valley; and the pass from Osh to Samarkand through Ferghana Valley (Williams, 2014).

The exchange and trade of livestock animals was one of the important elements of functioning of the West-East networks (Taylor et al., 2018). For example, horses raised in the Ferghana Valley were highly valued and were transported from Central Asia to China at the end of the first millennium BCE (Sinor, 1972; Taylor et al., 2018). According to Spengler (Spengler, 2015), caravans along with goods brought sheep, goat, and cattle into the mountains by the late third millennium, while Stevens et al. (Stevens et al., 2016) considered that sheep and taurine cattle reached China in 2000–1900 BC (Stevens et al., 2016). The latest zooarchaeological and genomic studies provided evidence that pastoralists inhabited the Alay Valley by the early Bronze Age, during the late 3rd millennium BCE, and sheep were the basis of this ancient herding society (Taylor et al., 2018). Considering this assumption, the predominant genetic pattern in the Alai breed (violet color) (K = 12, 24, **Figure 6**) possibly represents the ancestral authentic genomic component of the sheep inhabiting the Alai Valley. In this regard, the Alai sheep most likely is the most valuable national sheep resource in the Kyrgyzstan.

In addition, there was also an assumption that Mongolian tribes brought local fat-tailed sheep to the territory of Kazakhstan and Kyrgyzstan (Lushikhin, 1964). The Mongol invasion in China (Zhao et al., 2017) and in Central Asia including the Kyrgyzstan dated to the 12^th^ to 13^th^ centuries CE (Biran, 2004; Biran, 2013). As there were no genotyping data of Mongol sheep breeds, we were not able to address this assumption genetically. However, considering the detection of the same pattern of ancestry in the sheep breeds of the Kyrgyzstan, China, Iran, and partly India at K = 5 (**Figure 6**), admixture most likely took place before the Mongol conquerors came to Central Asia.

## Conclusion

Using the SNP genotyping data, we investigated for the first time the genetic diversity in the Kyrgyz sheep breeds. We found comparatively low levels of inbreeding, estimated by several methods, in the studied breeds. Nevertheless, conservation programs and rational management systems are required for the Alai and Kyrgyz coarse wool breeds that had small effective population sizes.

Our investigation revealed the presence of two ancestry patterns in the five sheep breeds from the Kyrgyz Republic. The Tien-Shan breed was the most differentiated within the group of the Kyrgyz sheep. The combination of our data with a worldwide and Russian sheep SNP set provided new insights into the origin and phylogenetic relations of the Kyrgyz sheep breeds.

Thus, our results suggest shared ancestry of the Kyrgyz coarse wool, Aykol and Gissar breeds with the Iranian, Chinese and Russian local fat-tailed and fat-rumped coarse wool sheep, while the Alai had a different genetic background that probably corresponds to the origin of ancient sheep inhabiting the Allay Valley. In addition, the Tien-Shan demonstrated a prevalence of European genetic background.

Our findings agreed with the documented facts on the creation of the studied breeds and mainly overlapped with archeological, historical, and genomic studies.

Present work addressed the genome-wide diversity and population structure of sheep breeds that originated in Central Asia for the first time and contributed to a better understanding of complex genetic connections and expected common history of the sheep breeds in Eurasia.

## Data Availability Statement

The datasets generated for this study can be found in Dryad https://doi:10.5061/dryad.37pvmcvff.

## Ethics Statement

The reported study was performed in accordance with the ethical guidelines of the L.K. Ernst Federal Science Center for Animal Husbandry. The protocol was approved by the Commission on the Ethics of Animal Experiments of the L.K. Ernst Federal Science Center for Animal Husbandry. The animal tissue samples were collected by trained personnel under strict veterinary rules in accordance with the Rules for conducting of laboratory research (tests) in the implementation of the veterinary control (supervision) approved by Council Decision Eurasian Economic Commission № 80 (November 10, 2017).

## Author Contributions

NZ and GB designed the study and participated in project coordination. EL and AZ collected the samples. TD and HR conducted the molecular genetic work. AD, ASh, HR, AS, EK, and IM processed the molecular genetic data. TD, AD, JS, HR, KW, NK, EL, GB, and NZ analyzed and discussed the data. TD, NZ, EL, and EK helped to draft the manuscript. TD wrote the final manuscript. All authors read and approved the final manuscript.

## Funding

The 50k SNP genotypes of the studied breeds were obtained within the framework of the Ministry of Science and Higher Education of Russia theme No. 0445-2019-0026 (АААА-А18-118021590138-1). The 600k SNP genotypes of the studied sheep breeds were obtained with financial support by the Russian Scientific Foundation (RSF) within Project No. 19-16-00070. The authors declare that the RSF and the Ministry of Science and Higher Education of Russia financed the study and did not have any influence on the results and their interpretation.

## Conflict of Interest

The authors declare that the research was conducted in the absence of any commercial or financial relationships that could be construed as a potential conflict of interest.
