# Supplementary figures and images for: Population Structure and Genetic Diversity of Sheep Breeds in the Kyrgyzstan
Source: Front Genet. 2019 Dec 12;10:1311. doi: 10.3389/fgene.2019.01311 (PMC6922024; doi:10.3389/fgene.2019.01311)

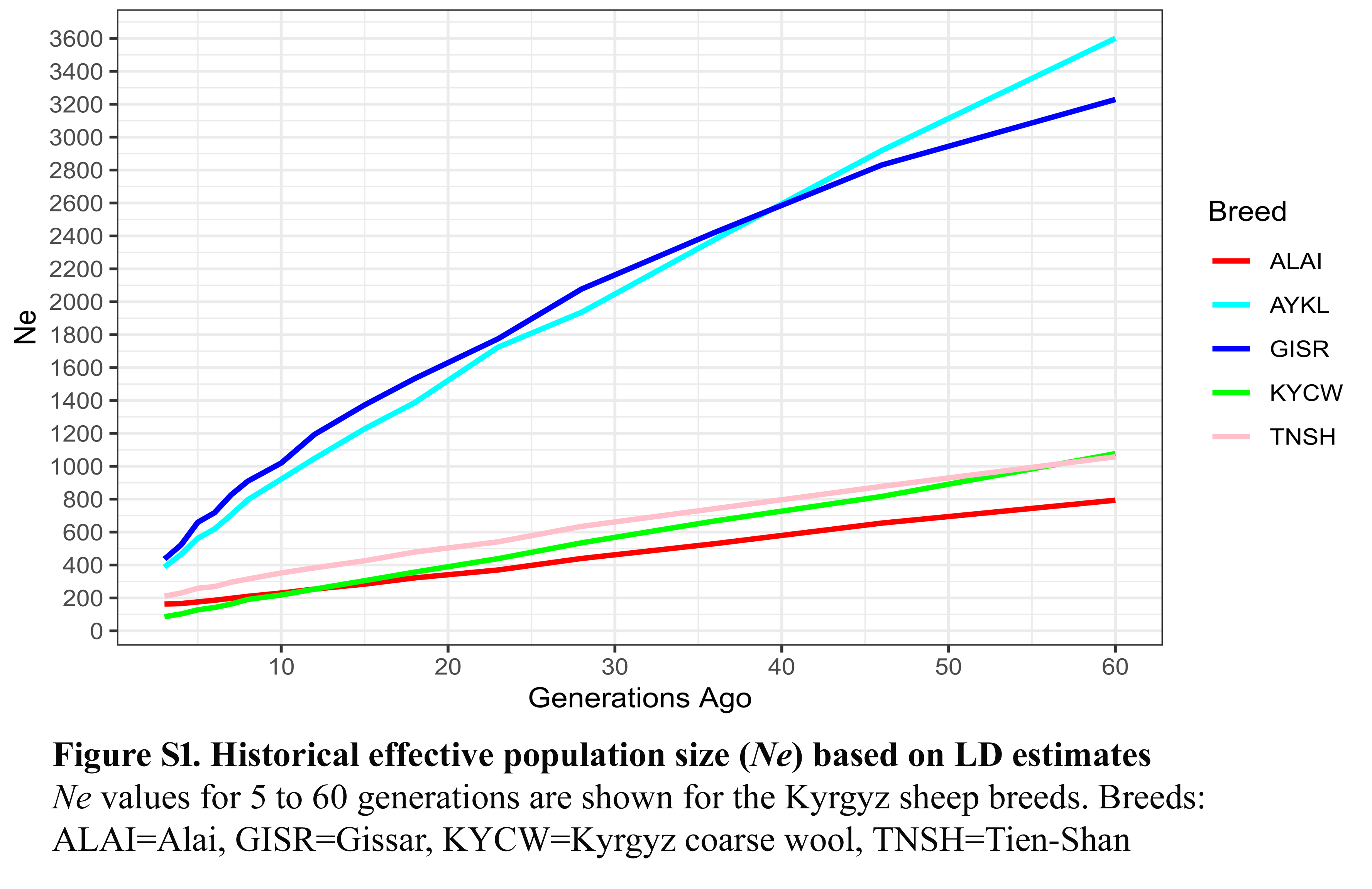

Supplement: Supplementary file 1 [file Image_1.tif]

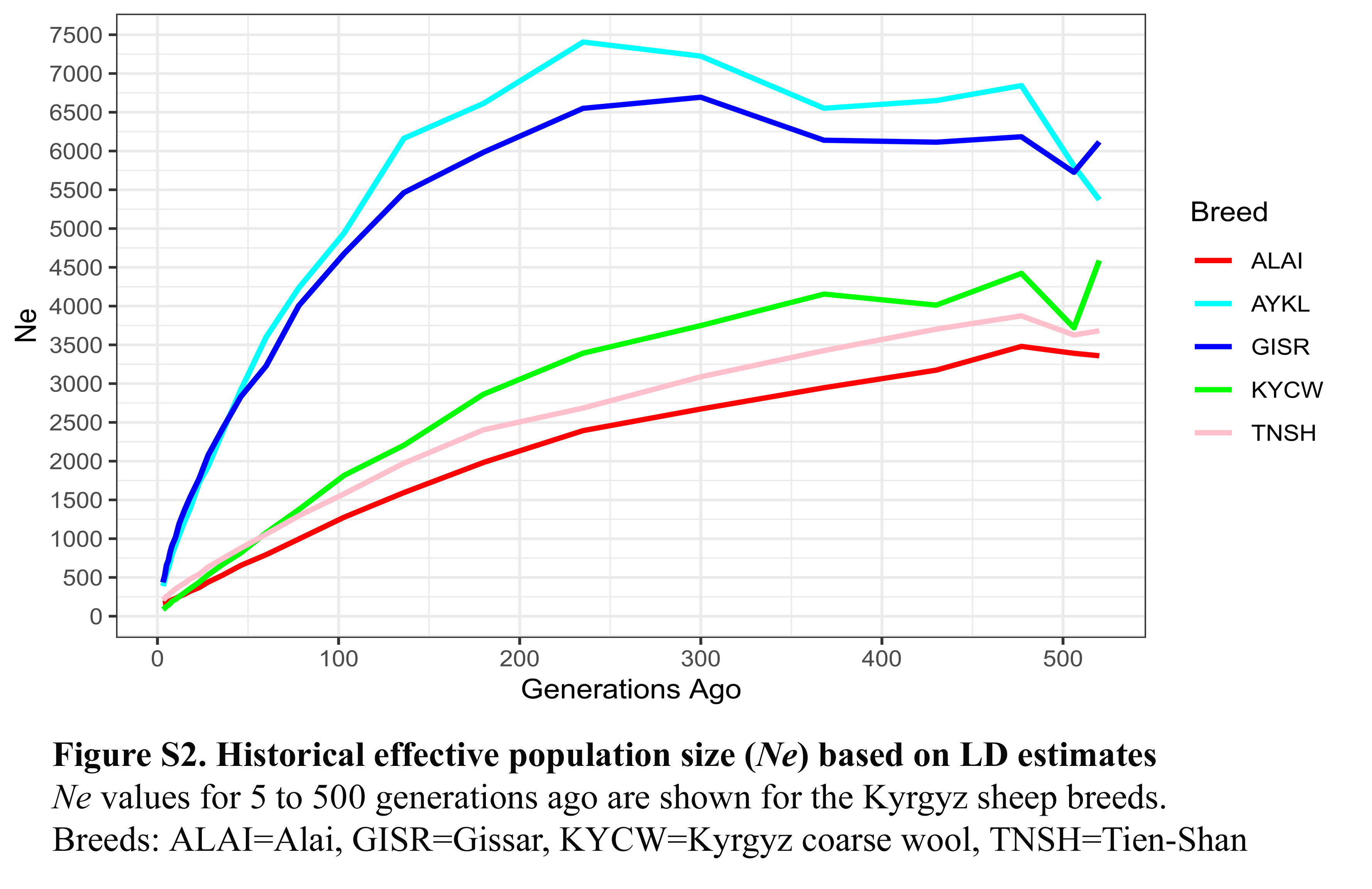

Supplement: Supplementary file 2 [file Image_2.tif]

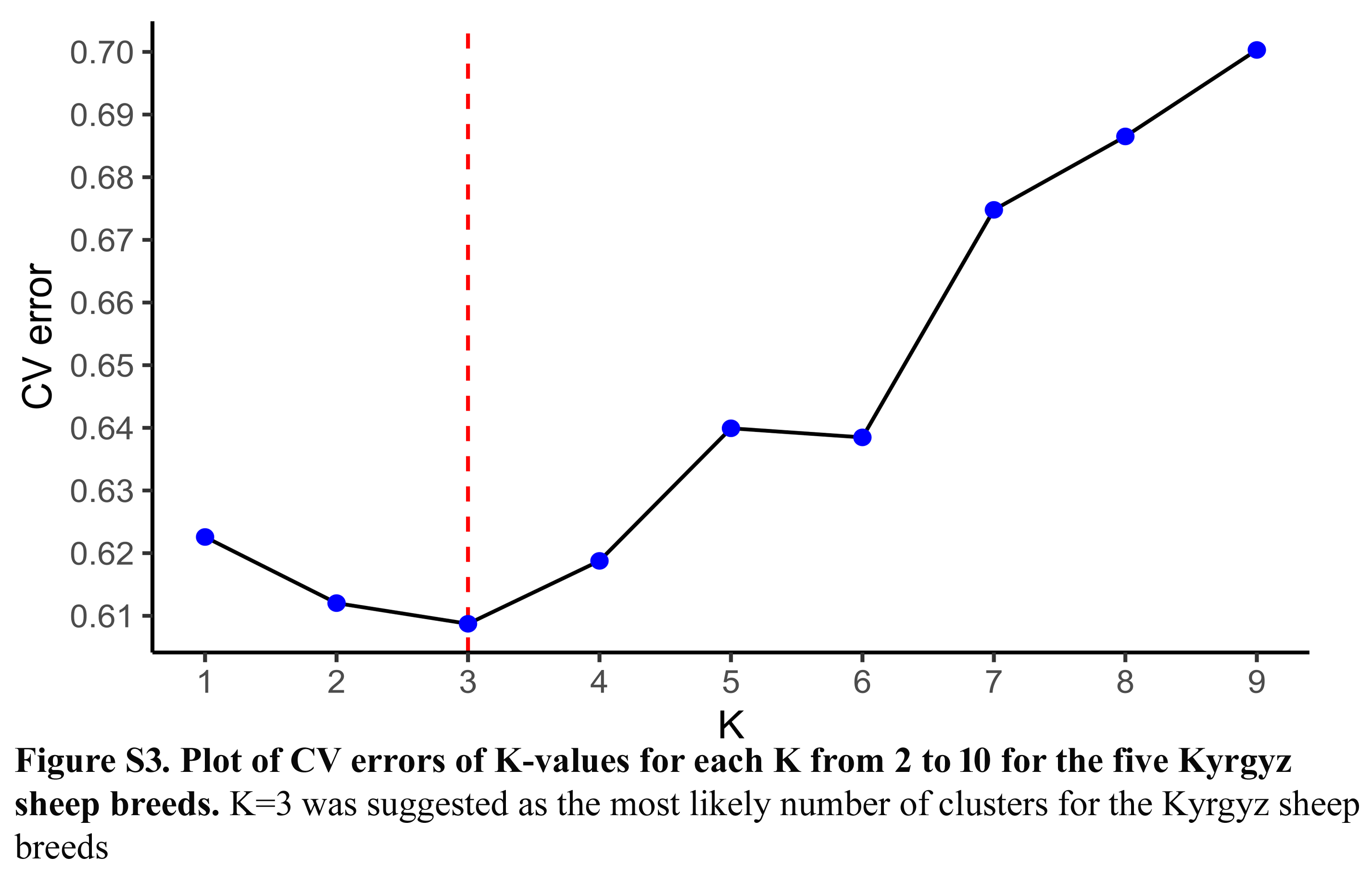

Supplement: Supplementary file 3 [file Image_3.tif]

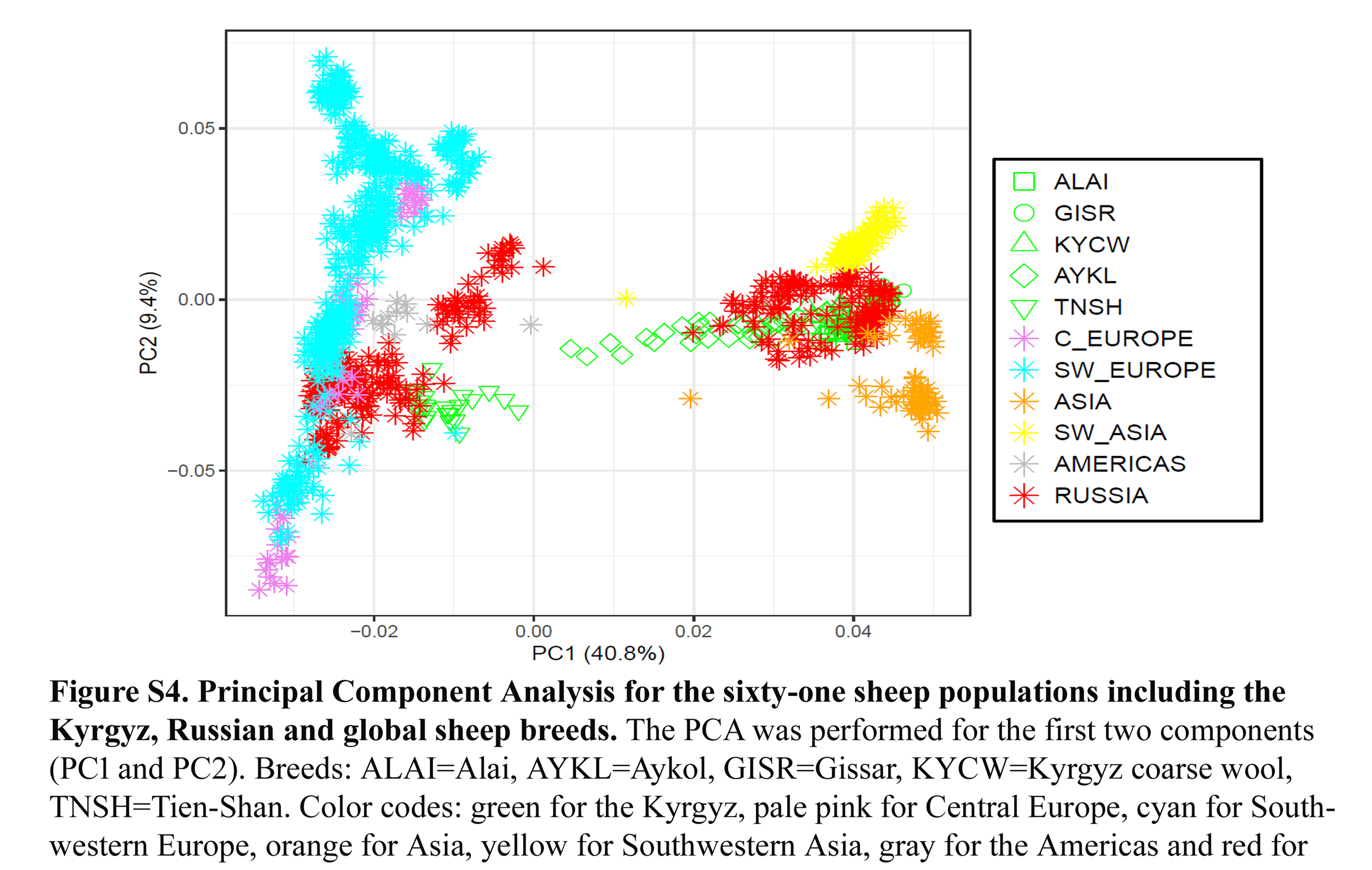

Supplement: Supplementary file 4 [file Image_4.tif]

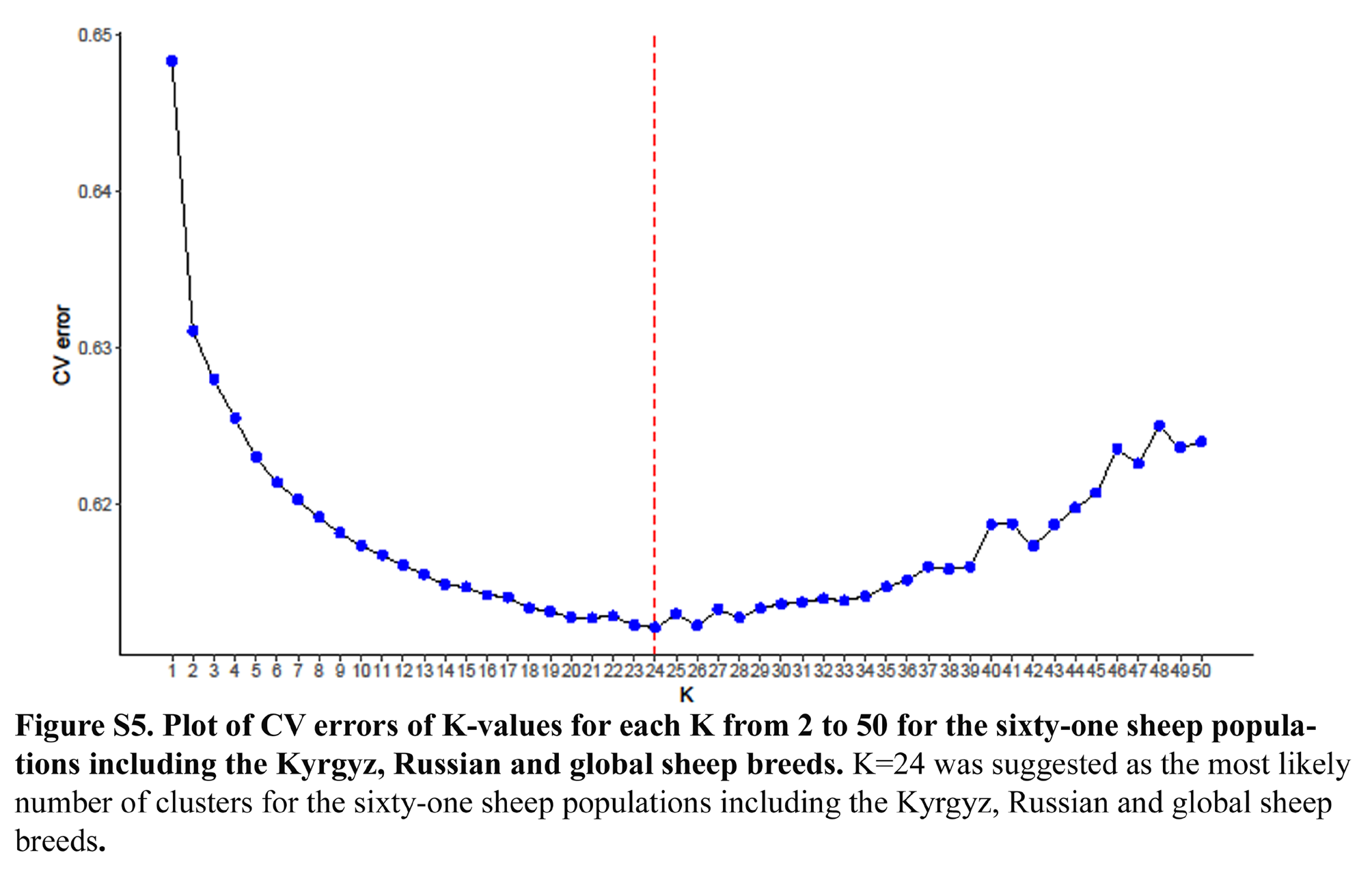

Supplement: Supplementary file 5 [file Image_5.tif]
