# Supplementary material for: Population Structure and Genetic Diversity of Sheep Breeds in the Kyrgyzstan
Source: Front Genet. 2019 Dec 12;10:1311. doi: 10.3389/fgene.2019.01311 (PMC6922024; doi:10.3389/fgene.2019.01311)
Supplement: Supplementary file 7 [file Table_2.docx]

**Table S2.** **Distribution of total number of ROH according to ROH categories across the Kyrgyz sheep breeds**

| ROH category | Alai | Aykol | Gissar | Kyrgyz  coarse wool | Tien-Shan |
| --- | --- | --- | --- | --- | --- |
| 1-2 | 2068 | 1841 | 2330 | 865 | 1413 |
| 2-4 | 256 | 133 | 200 | 58 | 245 |
| 4-8 | 139 | 12 | 24 | 5 | 93 |
| 8-16 | 72 | 9 | 8 | 4 | 21 |
| >16 | 39 | 2 | - | 6 | 2 |
