# Supplementary material for: Population Structure and Genetic Diversity of Sheep Breeds in the Kyrgyzstan
Source: Front Genet. 2019 Dec 12;10:1311. doi: 10.3389/fgene.2019.01311 (PMC6922024; doi:10.3389/fgene.2019.01311)
Supplement: Supplementary file 8 [file Table_3.docx]

**Table S3.** **The percentage of ROH according to ROH categories across the Kyrgyz sheep breeds**

| ROH category | Alai | Aykol | Gissar | Kyrgyz coarse wool | Tien-Shan |
| --- | --- | --- | --- | --- | --- |
| 1-2 | 80,34 | 92,19 | 90,94 | 92,22 | 79,65 |
| 2-4 | 9,95 | 6,66 | 7,81 | 6,18 | 13,81 |
| 4-8 | 5,40 | 0,60 | 0,94 | 0,53 | 5,24 |
| 8-16 | 2,80 | 0,45 | 0,31 | 0,43 | 1,18 |
| >16 | 1,52 | 0,10 | - | 0,64 | 0,11 |
