# Supplementary material for: Population Structure and Genetic Diversity of Sheep Breeds in the Kyrgyzstan
Source: Front Genet. 2019 Dec 12;10:1311. doi: 10.3389/fgene.2019.01311 (PMC6922024; doi:10.3389/fgene.2019.01311)
Supplement: Supplementary file 9 [file Table_4.docx]

**Table S4.** **Distribution of total number of ROHs across the chromosome in the Kyrgyz sheep breeds**

| OAR | Alai | Aykol | Gissar | Kyrgyz coarse wool | Tien-Shan |
| --- | --- | --- | --- | --- | --- |
| 1 | 260 | 185 | 291 | 120 | 176 |
| 2 | 257 | 224 | 235 | 108 | 173 |
| 3 | 204 | 178 | 195 | 79 | 176 |
| 4 | 122 | 90 | 111 | 40 | 87 |
| 5 | 114 | 91 | 98 | 39 | 83 |
| 6 | 157 | 108 | 127 | 50 | 100 |
| 7 | 113 | 74 | 117 | 47 | 65 |
| 8 | 84 | 55 | 69 | 33 | 49 |
| 9 | 105 | 89 | 100 | 27 | 65 |
| 10 | 100 | 104 | 136 | 45 | 70 |
| 11 | 72 | 72 | 71 | 24 | 53 |
| 12 | 80 | 55 | 75 | 33 | 47 |
| 13 | 112 | 92 | 107 | 38 | 85 |
| 14 | 88 | 53 | 78 | 26 | 44 |
| 15 | 76 | 62 | 76 | 33 | 67 |
| 16 | 64 | 48 | 83 | 22 | 49 |
| 17 | 85 | 63 | 93 | 23 | 67 |
| 18 | 75 | 52 | 62 | 28 | 47 |
| 19 | 63 | 52 | 66 | 20 | 37 |
| 20 | 48 | 34 | 55 | 13 | 26 |
| 21 | 59 | 34 | 48 | 16 | 36 |
| 22 | 44 | 41 | 63 | 16 | 28 |
| 23 | 78 | 40 | 85 | 27 | 49 |
| 24 | 46 | 39 | 50 | 13 | 32 |
| 25 | 32 | 25 | 24 | 5 | 33 |
| 26 | 36 | 37 | 47 | 13 | 30 |
| Total | 2574 | 1997 | 2562 | 938 | 1774 |
