# Supplementary material for: Population Structure and Genetic Diversity of Sheep Breeds in the Kyrgyzstan
Source: Front Genet. 2019 Dec 12;10:1311. doi: 10.3389/fgene.2019.01311 (PMC6922024; doi:10.3389/fgene.2019.01311)
Supplement: Supplementary file 10 [file Table_5.docx]

**Table S5. The percentage of chromosome residing in runs of homozygosity (ROH) per breed.**

| OAR | Alai | Aykol | Gissar | Kyrgyz coarse wool | Tien-Shan |
| --- | --- | --- | --- | --- | --- |
| 1 | 10.10 | 9.26 | 11.36 | 12.79 | 9.92 |
| 2 | 9.98 | 11.22 | 9.17 | 11.51 | 9.75 |
| 3 | 7.93 | 8.91 | 7.61 | 8.42 | 9.92 |
| 4 | 4.74 | 4.51 | 4.33 | 4.26 | 4.90 |
| 5 | 4.43 | 4.56 | 3.83 | 4.16 | 4.68 |
| 6 | 6.10 | 5.41 | 4.96 | 5.33 | 5.64 |
| 7 | 4.39 | 3.71 | 4.57 | 5.01 | 3.66 |
| 8 | 3.26 | 2.75 | 2.69 | 3.52 | 2.76 |
| 9 | 4.08 | 4.46 | 3.90 | 2.88 | 3.66 |
| 10 | 3.89 | 5.21 | 5.31 | 4.80 | 3.95 |
| 11 | 2.80 | 3.61 | 2.77 | 2.56 | 2.99 |
| 12 | 3.11 | 2.75 | 2.93 | 3.52 | 2.65 |
| 13 | 4.35 | 4.61 | 4.18 | 4.05 | 4.79 |
| 14 | 3.42 | 2.65 | 3.04 | 2.77 | 2.48 |
| 15 | 2.95 | 3.10 | 2.97 | 3.52 | 3.78 |
| 16 | 2.49 | 2.40 | 3.24 | 2.35 | 2.76 |
| 17 | 3.30 | 3.15 | 3.63 | 2.45 | 3.78 |
| 18 | 2.91 | 2.60 | 2.42 | 2.99 | 2.65 |
| 19 | 2.45 | 2.60 | 2.58 | 2.13 | 2.09 |
| 20 | 1.86 | 1.70 | 2.15 | 1.39 | 1.47 |
| 21 | 2.29 | 1.70 | 1.87 | 1.71 | 2.03 |
| 22 | 1.71 | 2.05 | 2.46 | 1.71 | 1.58 |
| 23 | 3.03 | 2.00 | 3.32 | 2.88 | 2.76 |
| 24 | 1.79 | 1.95 | 1.95 | 1.39 | 1.80 |
| 25 | 1.24 | 1.25 | 0.94 | 0.53 | 1.86 |
| 26 | 1.40 | 1.85 | 1.83 | 1.39 | 1.69 |
